# Metabolic challenges of glucose and lipid dysregulation in psoriatic arthritis: a narrative review from pathogenesis to clinical practice

**DOI:** 10.1007/s00592-025-02565-5

**Published:** 2025-08-14

**Authors:** Mauro Fatica, Sara Ferrigno, Eneida Çela, Arianna D’Antonio, Paola Conigliaro, Marina Cardellini, Susanna Longo, Massimo Federici, Maria Sole Chimenti

**Affiliations:** 1 Rheumatology, Allergology and Clinical Immunology, Department of Systems Medicine, University of Rome Tor Vergata, 00133 Rome, Italy; 2 Department of Systems Medicine, University of Rome Tor Vergata, Via Montpellier 1, 00133 Rome, Italy; 3 Center for Atherosclerosis, Policlinico Tor Vergata, Viale Oxford 81, 00133 Rome, Italy

**Keywords:** Psoriatic arthritis, Immunometabolism, Proinflammatory cytokines, Glucose metabolism, Lipid metabolism, Cardiometabolic risk

## Abstract

Emerging evidence highlights a complex interconnection between metabolic dysfunction and chronic inflammation in psoriatic arthritis (PsA), positioning glucose and lipid abnormalities not only as comorbidities but as active contributors to disease pathogenesis. In fact, rather than being incidental findings, conditions such as insulin resistance, atherogenic dyslipidemia, and visceral obesity interact with the immune system, amplifying inflammatory circuits and promoting joint and systemic damage. This review delves into the metabolic dimension of PsA, shedding light on how pro-inflammatory cytokines and adipokine imbalances reshape glucose and lipid homeostasis. Special attention is given to molecular pathways, such as impaired insulin signaling and altered lipid uptake, that link systemic inflammation to cardiometabolic risk. Furthermore, we examine the impact of these metabolic alterations in clinical practice and how antirheumatic therapies might improve metabolic balance and promote long-term cardiovascular protective effects. By unraveling these interactions, we aim to provide new insights into the clinical management of PsA and underscore the need for integrated therapeutic strategies that address both inflammation and metabolic health.

**Supplementary Information:**

The online version contains supplementary material available at 10.1007/s00592-025-02565-5.

## Introduction

Metabolic alterations are recognized as pivotal drivers of heightened autoimmunity and persistent inflammation across a range of rheumatic disorders [1]. Psoriatic arthritis (PsA) is a chronic inflammatory disease with articular and extra-articular involvement, often linked to metabolic comorbidities like dyslipidemia, insulin resistance (IR), and obesity, especially in male patients [2]. These metabolic derangements are not passive consequences of chronic inflammation but actively contribute to immune dysregulation and sustain a pro-inflammatory environment, driving disease progression in PsA through shared pathways linking systemic inflammation with metabolic dysfunction, atherosclerosis, and oxidative stress [3]. In fact, given the growing intersection between immune-mediated inflammation and metabolic dysfunction, PsA offers a relevant clinical model for better understanding how chronic inflammation drives glycemic and lipid abnormalities. This is particularly important in shared care models where rheumatologists and diabetologists jointly manage cardiometabolic risk in these patients. Therefore, this review explores the intricate interplay between glucose and lipid metabolism in PsA, emphasizing their mechanistic roles in immunopathogenesis and clinical manifestations, with a particular focus on the effects of pro-inflammatory cytokines and alterations in circulating adipokines. Additionally, the review assesses how these metabolic alterations impact the clinical management of patients with PsA and how antirheumatic therapies might restore glucose and lipid homeostasis and improve cardiometabolic health.

## Mechanisms of inflammation-induced glycemic changes in PsA

### Pro-inflammatory cytokines

Tumor necrosis factor alpha (TNF-α) may promote IR mainly by reducing tyrosine phosphorylation of the insulin receptor and insulin receptor substrate 1 (IRS-1), while increasing serine phosphorylation of IRS-1. This modification inhibits insulin receptor signaling, particularly in adipocytes and skeletal muscle cells, disrupting phosphoinositide 3-kinase (PI3K)/Akt activation and preventing glucose transporter type 4 (GLUT4) translocation to the cell membrane, thus impairing insulin signaling [4]. TNF-α also directly downregulates the expression of GLUT4, essential for insulin-mediated glucose uptake, and stimulates the synthesis of suppressor of cytokine signaling 3 (SOCS-3), an inhibitor of the insulin signaling pathway [5]. Moreover, TNF-α inhibits the synthesis of peroxisome proliferator-activated receptor gamma (PPARγ), a key transcription factor involved in adipogenesis and insulin sensitivity [6]. Finally, TNF-α increases fat mass and stimulates lipolysis in adipocytes, raising circulating free fatty acids (FFA), which interfere with insulin signaling [7].

**Fig. 1:**
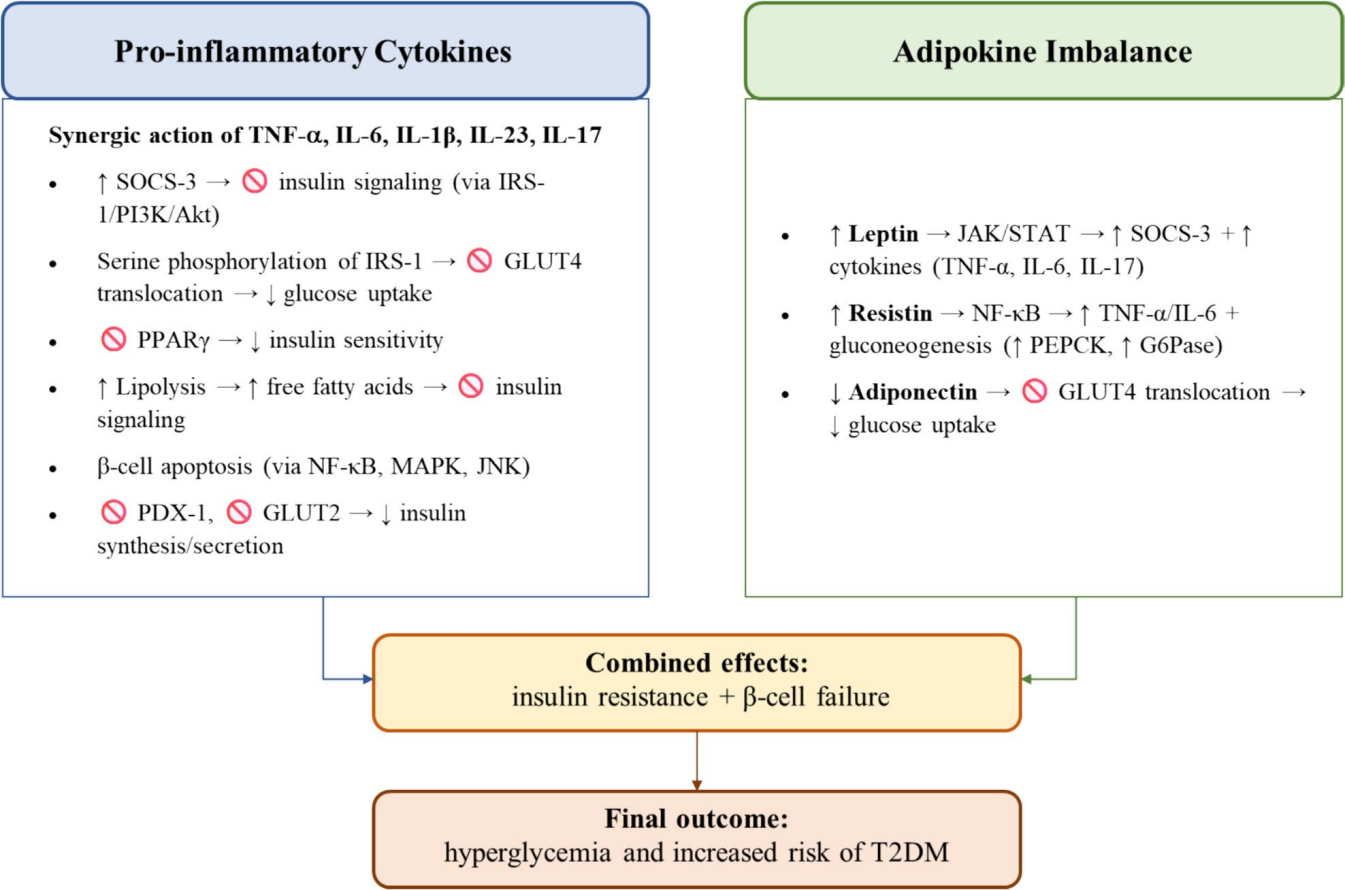
The mechanistic role of pro-inflammatory cytokines and adipokine imbalance on glycemic metabolism alterations in psoriatic arthritis (PsA). *TNF-α*, tumor necrosis factor alpha; *IL*, interleukin; *SOCS-3*, suppressor of cytokine signaling 3; *IRS-1*, insulin receptor substrate 1; *PI3K*, phosphoinositide 3-kinase; *GLUT4*, glucose transporter type 4; *PPARγ*, peroxisome proliferator-activated receptor gamma; *FFA*, free fatty acids; *NF-κB*, nuclear factor kappa-light-chain-enhancer of activated B cells; *MAPK*, mitogen-activated protein kinase, *JNK*, c-Jun N-terminal kinase; *PDX-1*, pancreatic duodenal homeobox-1; *GLUT-2*, glucose transporter type 2; *JAK/STAT*, Janus kinase/signal transducers and activators of transcription; *PEPCK*, phosphoenolpyruvate carboxykinase; *G6Pase*, glucose-6-phosphatase; *T2DM*, type II diabetes mellitus

The effect of interleukin (IL)-6 effect on glucose metabolism is dual and context-dependent, with chronic exposure inducing IR and acute increases enhancing insulin sensitivity. In PsA, chronic high IL-6 levels impair insulin signaling by altering IRS-1 phosphorylation and inducing SOCS-3 expression, similar to TNF-α [8]. IL-1β has a bimodal effect on pancreatic cells: low concentrations promote insulin release, while high, chronic concentrations in pro-inflammatory contexts induce β-cell apoptosis via Nuclear factor kappa-light-chain-enhancer of activated B cells (NF-κB), mitogen-activated protein kinase (MAPK), and c-Jun N-terminal kinase (JNK) pathways, increasing nitric oxide and reactive oxygen species production [9]. IL-23 and IL-17 enhance the expression of SOCS-3, impair PI3K and Akt attivation, and promote mitochondrial dysfunction and oxidative stress, which activate JNK, which then phosphorylates IRS-1 at serine residues, disrupting insulin signaling [10]. These cytokines act synergistically to impair pancreatic β-cell function by downregulating GLUT2 expression, the primary glucose transporter in β-cells, thereby reducing glucose uptake and insulin secretion. Moreover, IL-1β and TNF-α induce oxidative stress, contributing to β-cell dysfunction by impairing insulin synthesis, secretion, and survival. They also inhibit insulin gene transcription by suppressing pancreatic duodenal homeobox-1 (PDX-1), a key transcription factor for insulin production [11]. This ultimately results in reduced glucose uptake by β-cells, decreased insulin production, and increased apoptosis, markedly contributing to the development of hyperglycemia and an elevated risk of type II diabetes mellitus (T2DM) These mechanisms are summarized in Fig. 1.

### Adipokines imbalance

High leptin levels in PsA activate the Janus kinase/signal transducers and activators of transcription (JAK/STAT) pathway, leading to the upregulation of SOCS-3, impairing GLUT4-mediated glucose uptake, and enhance the production of TNF-α, IL-6, and IL-17, which further exacerbate IR [12]. Resistin levels are significantly elevated in PsA, activating NF-κB signaling and increasing TNF-α and IL-6 production. This disrupts insulin signaling by impairing IRS-1 function, reducing Akt phosphorylation, and ultimately inhibiting GLUT4 translocation and glucose uptake [13]. Moreover, resistin induces gluconeogenesis in the liver by upregulating phosphoenolpyruvate carboxykinase (PEPCK) and glucose-6-phosphatase (G6Pase), increasing blood glucose levels, and decreases hepatic insulin sensitivity, contributing to systemic insulin resistance [14]. PsA is associated with reduced adiponectin levels, an anti-inflammatory adipokine that enhances insulin sensitivity by promoting GLUT4 translocation and glucose uptake in muscle and adipose tissue, while also inhibiting pro-inflammatory cytokine production [15]. These mechanisms are summarized in Fig. 1.

Pro-inflammatory cytokines ( [], interleukin [IL]-6, IL-1β, IL-17, IL-23) and adipokine imbalance (↑ leptin, ↑ resistin, ↓ adiponectin) impair insulin signaling by inducing (), altering insulin receptor substrate 1 (IRS-1) phosphorylation, and inhibiting () translocation. This leads to insulin resistance, pancreatic β-cell dysfunction, and reduced insulin synthesis via inhibition of pancreatic duodenal homeobox-1 (PDX-1) and glucose transporter type 2 (GLUT2), ultimately contributing to hyperglycemia and type 2 diabetes mellitus (T2DM).

### Impact of glycemic changes in clinical practice

Due to the aforementioned molecular mechanisms, PsA significantly increases the risk of developing hyperglycemia and T2DM. In this context, the prevalence of T2DM in patients with PsA is notably higher than in the general population, ranging from 6 to 20% [16]. Additionally, hyperglycemia and T2DM further enhances the risk of developing other cardiometabolic comorbidities, including metabolic syndrome (MetS), non-alcoholic fatty liver disease (NAFLD), and major adverse cardiovascular events (MACE) [17]. Therefore, the presence of these conditions can significantly worsen clinical outcomes in PsA, as higher disease severity, greater radiographic progression, and reduced quality of life have been consistently observed in patients with cardiometabolic comorbidities [18].

Regarding pharmacological treatments, in PsA the use of corticosteroids is generally limited to intra-articular injections for the treatment of disease flares, particularly when only a few peripheral joints are involved [19]. Therefore, it is reasonable to infer that, in clinical practice, the impact of corticosteroids on glucose metabolism alterations is less pronounced in PsA than in other immune-mediatied rheumatic diseases. Regarding Non-Steroidal Anti-Inflammatory Drugs (NSAIDs), their use is generally intended to help control disease flares, making their administration typically short-term. While NSAIDs generally have a neutral impact on blood glucose levels, rare cases of hypoglycemia have been reported, likely due to their ability to increase insulin release from pancreatic β-cells by inhibiting ATP-sensitive potassium channels [20]. Notably, one study found that prolonged NSAIDs use is associated with a reduced risk of developing T2DM [hazard ratio (HR) 0.31, 95% CI, 0.26–0.36] [21]. Therefore, considering the uncommon occurance of hypoglycemic episodes and the usually short-term use of NSAIDs in PsA, they represent a generally safe treatment option even for patients with concomitant alterations in glucose homeostasis.

Each disease-modifying antirheumatic drug (DMARD), by controlling chronic inflammation through distinct pharmacokinetic mechanisms, has the potential to improve glucose metabolism in patients with PsA or at least prevent the worsening of pre-existing glycemic homeostasis alterations. However, only a few studies in the literature have specifically evaluated this aspect in a clinical setting. For instance, in a study conducted on 27 PsA patients, it was observed that methotrexate (MTX) did not significantly increase fasting plasma glucose (FPG) or hemoglobin A1c (HbA1c) levels in PsA patients over a 12-week observation period [22]. Similarly, in a retrospective study on a large cohort of patients with rheumatological diseases, it was found that the use of MTX in patients with psoriasis (PsO) or PsA had a protective effect against the development of T2DM, with an adjusted HR of 0.35 (95% CI 0.28–0.43) [23]. Regarding other csDMARDs, to the best of our knowledge there are no studies specifically conducted on PsA patients in this regard. However, neither leflunomide nor sulfasalazine appear to impair glucose metabolism significantly, and are safely used in patients with concomitant T2DM [24].

As for biologic drugs, the most robust data are on anti-TNF-α agents. Several studies have shown that various anti-TNF-α agents have a generally neutral effect on FPG up to 6 months after starting treatment, with etanercept even being reported to reduce FPG in obese patients with MetS [25]. Additionally, a retrospective PsO cohort analysis found that patients on TNF-α antagonists had a lower risk of developing new-onset T2DM compared to those on non-biologic DMARDs (HR 0.62, 95% CI 0.42–0.91) [26]. Regarding bDMARDs targeting the IL-23/IL-17 axis, real-life data demonstrate a neutral effect of these drugs on glucose homeostasis [17].

Considering tsDMARDs, the phosphodiesterase-4 (PDE4) inhibitor apremilast has been shown to significantly reduce HbA1c levels after 16 weeks of treatment, particularly in PsA patients whose baseline HbA1c was above 6.5%, regardless of whether they were on antidiabetic drugs [27]. Although JAK inhibitors show promise in the treatment of MetS, and some studies have been conducted on PsA patients with concomitant MetS treated with these agents, robust data specifically examining their effects on glucose metabolism in real-life settings are currently lacking [28, 29]. As a result, the impact of these drugs on glycemic control in PsA patients still needs to be fully evaluated.

Finally, there is evidence in the literature suggesting that MetS may reduce the effectiveness of certain medications, particularly anti-TNF-α agents [24]. In this context, the reduced effectiveness of these drugs appears to be primarily attributed not to pre-existing glucose metabolism alterations, but rather to the presence of obesity, which may impair therapeutic response through mechanisms such as altered pharmacokinetics and sustained chronic inflammation [30]. These key aspects are summarized in Supplementary Table 1.

## Mechanisms of inflammation-induced lipidic changes in PsA

### Pro-inflammatory cytokines

TNF-α and IL-6 suppress the expression and activity of lipoprotein lipase (LPL), a key enzyme responsible for hydrolyzing TG in circulating chylomicrons and very low-density lipoproteins (VLDL), thereby facilitating the uptake of FFA by peripheral tissues [31, 32]. The suppression of LPL activity leads to impaired TG clearance and contributes to the development of hypertriglyceridemia. These cytokines also promote hepatic lipogenesis and impair fatty acid (FA) oxidation, increasing the risk of NAFLD [33].

TNF-α and IL-17 promote the formation of oxidized low-density lipoproteins (oxLDL), which enhances vascular inflammation [34]. Moreover, the cathelicidin LL37, overexpressed in psoriatic skin, binds self-DNA to induce type I interferons and IL-17 production, while promoting LDL uptake in macrophages via LDL receptor (LDLR), scavenger receptor B1 (SR-B1), and CD36, leading to cholesterol accumulation and atherosclerosis, and linking psoriatic inflammation to increased CV risk [35].

**Fig. 2:**
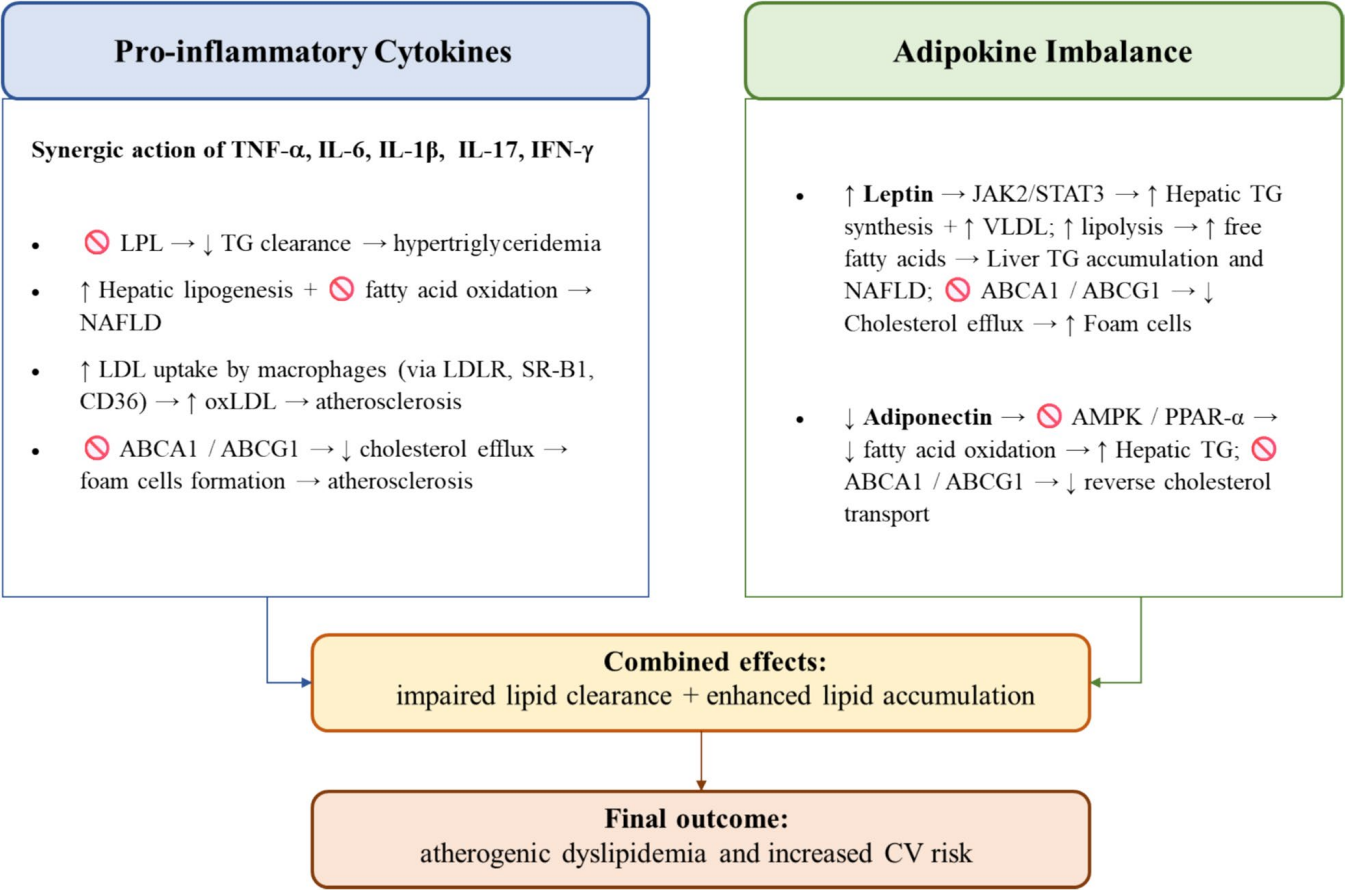
The mechanistic role of pro-inflammatory cytokines and adipokine imbalance on lipid metabolism alterations in psoriatic arthritis (PsA). *TNF-α*, tumor necrosis factor alpha; *IL*, interleukin; *IFN-γ*, interferon gamma; *LPL*, lipoprotein lipase; *TG*, triglycerides; *FA*, fatty acids; *NAFLD*, non-alcoholic fatty liver disease; *LDL*, low-density lipoproteins; *LDLR*, low-density lipoproteins receptor; *SR-B1*, scavenger receptor B1; *oxLDL*, oxydated low-density lipoproteins; *ABCA1/ABCG1*, ATP-binding cassette transporters A1 and G1; *JAK/STAT*, Janus kinase/signal transducers and activators of transcription; *VLDL*, very- low-density lipoproteins; *FFA*, free-fatty acids; *AMPK*, AMP-activated protein kinase; *PPARα*, peroxisome proliferator-activated receptor alpha; *CV*, cardiovascular

TNF-α, IL-1β, and IFN-γ impair cholesterol efflux from macrophages by downregulating ATP-binding cassette transporters A1 and G1 (ABCA1, ABCG1), key mediators of reverse cholesterol transport, reducing cholesterol clearance and leading to lipid accumulation within macrophages [36]. This fosters foam cell formation, thereby contributing to the increased CV risk observed in PsA patients. The resulting pro-atherogenic environment further exacerbates systemic inflammation, creating a vicious cycle between altered lipid metabolism and immune dysregulation [37]. These mechanisms are summarized in Fig. 2.

### Adipokines imbalance

Leptin contributes to dyslipidemia through multiple molecular mechanisms. It mainly promotes hepatic lipogenesis by activating the JAK2/STAT3 pathway, leading to increased TG synthesis and VLDL production [38]. Additionally, leptin enhances lipolysis in adipocytes via β-adrenergic receptor signaling, increasing FFA release into circulation. Excess FFAs are taken up by the liver, fueling hepatic TG accumulation and contributing to NAFLD [39]. Leptin also impairs reverse cholesterol transport by downregulating ATP-binding cassette transporters A1 and G1 (ABCA1, ABCG1) in macrophages, reducing cholesterol efflux and promoting foam cell formation, thereby exacerbating atherogenesis [12]. Adiponectin, an anti-inflammatory adipokine typically reduced in PsA, enhances hepatic fatty acid oxidation by activating AMP-activated protein kinase (AMPK) and peroxisome proliferator-activated receptor-α (PPAR-α), thereby reducing TG accumulation in the liver [40]. Lower adiponectin levels impair these pathways, leading to increased hepatic lipogenesis and VLDL secretion. Additionally, adiponectin promotes reverse cholesterol transport by upregulating ATP-binding cassette transporters A1 and G1 (ABCA1, ABCG1) in macrophages, facilitating cholesterol efflux and reducing foam cell formation [41]. These mechanisms are summarized in Fig. 2.

## Impact of lipid changes in clinical practice

The prevalence of dyslipidemia in PsA patients is variable but generally estimated at around 24%, occurring more frequently than in patients with isolated PsO and significantly higher than in the general population [42]. PsA patients exhibit more frequently than RA patients one or more features of MetS, including obesity and insulin resistance, which contribute to a distinctly atherogenic lipid profile characterized by elevated triglycerides (TG), decreased high-density lipoproteins (HDL) levels, and increased small, dense LDL particles [43]. In fact, the risk of MetS in PsA patients is significantly higher than in those with RA, with a reported hazard ratio of 1.66 ± 0.038 (95% CI 1.54–1.79) [44].

Most data on lipid alterations in PsA come from studies conducted on obese patients, who frequently present with dyslipidemia. Obesity significantly impacts PsA by exacerbating disease activity, accelerating radiographic progression, and reducing quality of life [45]. Excess adipose tissue promotes a chronic pro-inflammatory state that intensifies cytokine production, thereby worsening joint inflammation and damage, impairing treatment, and reducing overall quality of life [17]. Moreover, obese patients are significantly less likely to achieve and sustain remission or low disease activity over time [46].

As previously mentioned, obesity significantly impacts the effectiveness of pharmacological therapy, mainly in PsA, particularly TNF-α inhibitors, whereas drugs targeting the IL-23/IL-17 axis appear to be less affected by the presence of obesity [17]. As a broad observation, most anti-rheumatic treatments, by dampening systemic inflammation have effects on lipid metabolism and circulating lipid levels.

Corticosteroids can significantly influence lipid metabolism through various molecular mechanisms. They upregulate key lipogenic enzymes such as acetyl-CoA carboxylase and FA synthase, enhancing hepatic de novo lipogenesis and increasing VLDL production [47]. Additionally, glucocorticoids stimulate lipolysis in adipose tissue, elevating circulating FFA, which can be re-esterified in the liver, contributing to hepatic steatosis and dyslipidemia [48]. These alterations are mediated through both genomic pathways, involving glucocorticoid receptor-mediated gene transcription, and non-genomic mechanisms, affecting cellular signaling pathways. Consequently, long-term corticosteroid therapy may lead to unfavorable changes in lipid profiles, including elevated TG and LDL cholesterol levels, thereby increasing CV risk.​ These alterations typically develop in the context of long-term corticosteroid therapy, which is less commonly used in PsA than in RA [19]. However, given the higher prevalence of obesity and MetS in PsA, careful monitoring is essential for patients receiving these treatments, as corticosteroids contribute to an atherogenic lipid profile and may further increase CV risk in a setting of chronic inflammation.

NSAIDs have a relatively neutral or inconsistent effect on lipid profiles, and their potential CV impact is more closely related to pro-thrombotic and hypertensive effects rather than direct alterations in lipid metabolism [49]. Therefore, the use of NSAIDs should be carefully evaluated in patients with existing CV risk factors, despite their limited influence on lipid profiles.

The effects of csDMARDs on lipid metabolism in PsA patients have not been systematically evaluated through dedicated studies. However, in clinical practice, these agents are generally considered to have a neutral impact on lipid homeostasis and are safely used in patients with concomitant dyslipidemia or MetS [50, 51].

TNF-α inhibitors have been shown to reduce serum levels of Lipoprotein(a), a pro-inflammatory, atherogenic LDL-like complex composed of apolipoprotein(a) and apolipoprotein(b) [52]. Additionally, TNF inhibitors increase the levels of Apolipoprotein A-1, which has anti-inflammatory and anti-apoptotic properties. However, these beneficial effects are counterbalanced by an observed rise in TG and Apolipoprotein B levels, suggesting that the overall impact on the lipid profile in PsA patients remains uncertain [52]. In contrast, a recent study involving 165 patients with PsO found that treatment with TNF inhibitors, IL-23 inhibitors, and the IL-17A inhibitor ixekizumab resulted in a significant increase in HDL after 12 weeks, with less pronounced effects on LDL cholesterol and TG [53]. In a pooled analysis of data from two phase III studies and one ongoing long-term extension study, the treatment with oral Janus kinase (JAK) inhibitor Tofacitinib (5 or 10 mg twice daily) resulted in significant increases in low-density lipoprotein cholesterol (LDL-c) and HDL levels after 3 and 6 months. However, no significant changes in the LDL:HDL or total cholesterol (TC):HDL ratios were observed. Blood pressure remained stable over 24 months, and only 0.6% of patients experienced MACE [54]. This suggests that while tofacitinib increases serum lipid levels in PsA patients, the overall CV risk appears low, although long-term follow-up is still needed. A systematic review was conducted to evaluate the effect of upadacitinib, a JAK-1 selective inhibitor, on lipid profile and CV risk. Nineteen randomized controlled trials (RCTs) involving 10,656 patients (with RA, PsA, SpA or Crohn’s Disease) were included [55]. Upadacitinib (3–48 mg/day) increased both LDL-c and HDL-c in 15 studies, while the LDL-c:HDL-c ratio remained unchanged. No significant effect on MACE was observed, with a risk ratio (RR) of 0.62. These findings, although based on patients with various conditions and not solely PsA, align with the results seen with tofacitinib.

A prospective study on PsA and PsO patients treated with apremilast for six months showed a significant mean weight loss of 2.2 kg and BMI reduction of 0.8 kg/m^2^, primarily due to decreased abdominal subcutaneous fat, despite no significant changes were observed in lipid profile and glucose homeostasis [27]. Moreover, a retrospective study on patients with moderate-to-severe PsO treated with apremilast for up to 52 weeks showed potential long-term metabolic benefit of apremilast on weight and lipid profile: in particular, significant weight and BMI reduction, decreased TG levels at 24 and 52 weeks, and increased HDL at 52 weeks, with no significant changes in total cholesterol or LDL [56]. These key aspects are summarized in Supplementary Table 2.

## Assessment of glycemic and lipidic changes in PsA patients

In clinical practice, the assessment of glucose metabolism alterations in patients with rheumatic diseases is crucial, especially considering the potential impact of chronic inflammation and certain medications on glycemic control. The coexistence of inflammatory arthritis and T2DM worsens glucose control and increases the risk of vascular, renal, and infectious complications, especially relevant in the context of immunosuppressive therapy [57]. On the other hand, it has been observed that patients with T2DM, particularly females, have a higher predisposition to develop inflammatory arthritis [58]. While specific guidelines from the European Alliance of Associations for Rheumatology (EULAR) and the American College of Rheumatology (ACR) specifically regarding glucose monitoring in rheumatic patients are limited, general recommendations can be inferred from broader clinical guidelines. According to the American Diabetes association (ADA) Professional Practice Committee, the European Association for the Study of Diabetes (EASD) and the European Society of Cardiology (ESC), key screening tests include the fasting blood glucose (FBG), oral glucose tolerance test (OGTT), and determination of hemoglobin A1c (HbA1c) [59, 60].

Overall, screening for T2DM is recommended in individuals with known risk factors, such as obesity, family history of diabetes, hypertension, and dyslipidemia. Additionally, screening is recommended for patients planning a pregnancy or who are pregnant, starting from the first prenatal visit and continuing throughout gestation, particularly for those with known risk factors [59]. For adults with overweight or obesity and at least one additional risk factor, screening for prediabetes and T2DM is recommended regardless of age; for those without these risk factors, routine screening should begin at age 35 [61].

The metabolic score for insulin resistance (METS-IR) has demonstrated a stronger association with all-cause and cardiovascular mortality in the U.S. population compared to other IR indices, including the TG/HDL-C ratio, the triglyceride-glucose (TyG) index, and Homeostatic Model Assessment of Insulin Resistance (HOMA-IR) [62]. This correlation is especially pronounced in individuals under 65 years of age. In PsA, a baseline METS-IR value above 2.48 has been linked to an increased risk of cardiovascular events during follow-up [62].

A standard lipid profile—including TC, HDL, and TG—is routinely used to estimate plasma lipoprotein levels. According to the 2016 EULAR guidelines, TC and HDL should be assessed for cardiovascular risk stratification in RA and PsA, ideally during stable or remitted disease [63]. Lipid levels can be measured in either fasting or non-fasting states and, notably, the TC/HDL ratio is a more sensitive predictor of cardiovascular risk in RA than individual lipid indeces [63]. LDL cholesterol is typically calculated using the Friedewald formula, though this method has limitations, particularly in cases of high TG or non-fasting samples [64].

In patients with dyslipidemia, ApoB measurement is recommended for more precise risk assessment, while Lp(a) should be measured at least once in a lifetime to identify individuals at high lifetime CV risk. Neither EULAR nor ACR guidelines currently provide specific recommendations on the type or frequency of lipid monitoring [65]. In addition, for patients with inflammatory arthritis, such as RA and PsA, the EULAR recommends that a CV risk assessment should be performed at least once every five years, with reassessment following any significant changes in antirheumatic therapy [63].

The Systematic Coronary Risk Estimation (SCORE) system is widely used in Europe to estimate 10-year fatal CV risk and can be recalibrated for different populations. The SCORE2 algorithm is typically used for general risk assessment, while the SCORE2-OP and the SCORE2-Diabetes are specifically applied for individuals over 70 years of age or with concomitant T2DM, respectively [66–68]. These algorithms are currently used in apparently healthy individuals to estimate 10-year CV risk. Based on the obtained percentage result, patients are classified into very high, high, moderate, or low risk categories. Patients with established atherosclerotic CV disease are automatically classified into the very high-risk category. This classification forms the basis for initiating therapy targeting the main CV risk factors [65]. Since this score was developed for the general population, guidelines recommend applying a 1.5 correction factor for all RA patients, regardless of disease duration or extra-articular manifestations, to provide a more accurate risk assessment and guide targeted diagnostic and therapeutic strategies [63]. However, while this adjustment is established for RA, it remains unvalidated for PsA, and clinical trials specifically investigating CV risk treatment in these patients are still lacking.

To reduce CV risk in these patients, It is advisable to adopt lifestyle modifications including a healthy diet, regular exercise, and smoking cessation for all individuals [69]. Furthermore, optimal disease control should be pursued, and systemic corticosteroids should be tapered to the lowest effective dose or discontinued as soon as possible. In fact, EULAR guidelines for glucocorticoid therapy underscore the importance of monitoring potential adverse effects, including those related to glucose metabolism [70]. A summary of these clinical considerations is provided in Table 1.

**Table 1:** Summary of key clinical aspects in the assessment of glucose and lipid metabolism in psoriatic arthritis (PsA)

Domain	Key elements	Notes
Glucose metabolism screening	FBG, OGTT, HbA1c	ADA, EASD, ESC recommendations [59, 60]
	Screening advised in: obesity, family history, hypertension, dyslipidemia, pregnancy	ADA recommendations [59, 61]
	Begin routine screening at age 35 (if no other risk factors)	ADA recommendations [61]
Insulin resistance indices	METS-IR, TG/HDL ratio, TyG index, HOMA-IR	METS-IR shows strongest correlation with CV mortality [62]
	METS-IR > 2.48 linked to CV events in PsA	[62]
Lipid profile	TC, HDL, TG, LDL (via Friedewald formula)	TC/HDL ratio more predictive of CV risk in RA and PsA [63]
	ApoB and Lp(a) for refined risk stratification	Lp(a) to be measured at least once in life [65]
	Lipid levels can be measured in fasting or non-fasting states	Real-world evidence [63, 64]
CV risk assessment	SCORE2, SCORE2-OP, SCORE2-Diabetes algorithms	Used for 10-year CV risk estimation [66–68]
	RA: apply × 1.5 correction factor to SCORE	Validated only for RA, not PsA [63]
	CV risk assessment every 5 years or after treatment changes	EULAR recommendations [63]
Preventive strategies	Healthy diet, exercise, smoking cessation	General CV risk reduction [69]
	Taper corticosteroids to minimum dose	Monitor for glucose metabolism effects [70]
	Maintain tight disease control	Crucial for metabolic and CV risk

*FBG*, Fasting Blood Glucose; *OGTT*, Oral Glucose Tolerance Test; *HbA1c*, Hemoglobin A1c; *ADA*, American Diabetes association; *EASD*, European Association for the Study of Diabetes; *ESC*, European Society of Cardiology; *METS-IR*, Metabolic Score for Insulin Resistance (METS-IR); *TG*, Triglycerides; *HDL*, High-Density Lipoproteins; *TyG*, Triglyceride-Glucose; *HOMA-IR*, Homeostatic Model Assessment of Insulin Resistance; *TC*, Total Cholesterol; *LDL*, Low-Density Lipoproteins; *RA*, Rheumatoid Arthritis; *ApoB*, Apolipoprotein B; *Lp(a)*, Lipoprotein(a); *CV*, Cardiovascular; *SCORE*, Systematic Coronary Risk Estimation; *EULAR*, European Alliance of Associations for Rheumatology

## Conclusion

Alterations in glucose and lipid metabolism are commonly observed in patients with PsA, potentially complicating disease management. These metabolic disturbances arise through highly complex molecular mechanisms that are not yet fully understood, with chronic inflammation playing a central role in orchestrating these pathophysiological processes. Moreover, such metabolic impairments—particularly by increasing the risk of a broad spectrum of comorbidities—may significantly influence disease activity, structural damage progression, and therapeutic response. Conversely, several antirheumatic therapies have demonstrated benefical metabolic effects, largely through their capacity to control underlying inflammation. Although specific EULAR and ACR guidelines for metabolic monitoring in rheumatic patients are currently limited, existing general recommendations, combined with disease-specific considerations, provide a valuable framework for clinical practice.

Routine screening for abnormalities in glucose and lipid metabolism is particularly recommended in patients with established risk factors. However, such evaluation should not be assessed in isolation, but rather as integral components of a comprehensive cardiometabolic risk monitoring strategy. Monitoring approaches should be individualized, taking into account the patient’s treatment regimen, comorbidities, and level of disease activity. Special consideration should be given to patients with persistently high disease activity, despite multiple therapeutic interventions, who continue to exhibit signs of detectable inflammation through clinical evaluation, laboratory markers, or imaging studies [71]. In fact, these patients exemplify a clinical phenotype in which the persistence of chronic inflammation, even at a subclinical level, markedly heightens the risk of developing cardiometabolic comorbidities. These insights highlight the importance for diabetologists to recognize PsA not only as a rheumatologic condition but also as a model of inflammation-induced metabolic dysfunction. This awareness is crucial, particularly within shared care models where collaboration between specialists can optimize patient outcomes. Understanding these inflammation-driven metabolic changes also plays a key role in accurately stratifying CV risk, enabling more effective prevention and management strategies tailored to this high-risk population. However, continued research and future guideline updates are needed to establish more precise protocols tailored to the needs of these patients.

## Supplementary Information

Below is the link to the electronic supplementary material.

Supplementary file1 (DOCX 18 KB)
