# Supplementary material for: Metabolic challenges of glucose and lipid dysregulation in psoriatic arthritis: a narrative review from pathogenesis to clinical practice
Source: Acta Diabetol. 2025 Aug 14;62(11):1831–41. doi: 10.1007/s00592-025-02565-5 (PMC12640331; doi:10.1007/s00592-025-02565-5)
Supplement: Supplementary file 1 — Supplementary file1 (DOCX 18 KB) [file 592_2025_2565_MOESM1_ESM.docx]

| Aspect | Findings |
| --- | --- |
| Corticosteroids | Mostly used intra-articularly in PsA, therefore general minimal systemic impact on glucose metabolism [19]. |
| NSAIDs | Generally safe in PsA although rare cases of hypoglycemia reported [20]; long-term use might reduce T2DM risk [21]. |
| Methotrexate | No significant changes in FPG or HbA1c [22], long-term use might reduce T2DM risk [23]. |
| Other csDMARDs | Leflunomide, sulfasalazine: no major effect on glucose metabolism [24]. |
| Anti-TNF-α | Generally neutral on FPG, etanercept may reduce FPG in MetS [25]; long-term use might reduce T2DM risk [26]. |
| Anti-IL-17 / IL-23 | Neutral impact on glucose homeostasis in real-world data [17]. |
| Apremilast | Reduced HbA1c after 16 weeks, especially in patients with baseline HbA1c >6.5% [27]. |
| JAK inhibitors | Promising for MetS, insufficient real-life data in PsA for glycemic effects [28, 29]. |
| MetS and drug efficacy | May reduce efficacy of anti-TNF-α agents, mainly due to obesity-related factors (pharmacokinetics, persistent inflammation) [24, 30]. |

**Supplementary Table 1. Impact of glycemic changes and antirheumatic therapies in psoriatic arthritis (PsA).** Legend: T2DM, Type 2 Diabetes Mellitus; MetS, Metabolic Syndrome; NAFLD, Non-Alcoholic Fatty Liver Disease; MACE, Major Adverse Cardiovascular Events; NSAIDs, Non-Steroidal Anti-Inflammatory Drugs; FPG, Fasting Plasma Glucose; HbA1c, Hemoglobin A1c; csDMARDs, Conventional Synthetis Disease-Modifying Antirheumatic Drugs; TNF-α, Tumor Necrosis Factor Alpha; IL, Interleukin; JAK, Janus Kinase.

| Aspect | Findings |
| --- | --- |
| Impact of obesity | Worsens PsA by increasing disease activity, accelerating structural progression, and impairing quality of life; it also reduces treatment response, especially to TNF-α inhibitors [17, 45, 46]. |
| Corticosteroids | Long-term use may lead to elevated TG and LDL due to increased hepatic lipogenesis and circulating FFA [19, 47, 48]. |
| NSAIDs | Neutral impact on lipid metabolism, CV risk is more related to thrombosis and hypertension than lipid alterations [49]. |
| csDMARDs | Generally considered safe in patients with dyslipidemia or MetS and do not significantly alter lipid metabolism [50, 51]. |
| TNF-α Inhibitors | Reduce pro-atherogenic Lp(a) and increase anti-inflammatory ApoA-1 levels, but may also increase TG and ApoB, leading to an overall unclear lipid effect [52]. |
| IL-17/ IL-23 Inhibitors | Associated with increased HDL levels, with minor changes in LDL and TG after 12 weeks of treatment [53]. |
| JAK Inhibitors | Tofacitinib and upadacitinib raise both LDL and HDL, without significantly altering LDL:HDL or TC:HDL ratios, with stable blood pressure and low MACE incidence [54, 55]. |
| Apremilast | Potential benefits including reduced TG and increased HDL levels, alongside weight loss [56, 57]. |
|  |  |

**Supplementary Table 2. Impact of lipidic changes and antirheumatic therapies in psoriatic arthritis (PsA).** Legend: PsO, Psoriasis; TG, Triglycerides; HDL, High-Density Lipoproteins; LDL, Low-Density Lipoproteins; TNF-α, Tumor Necrosis Factor Alpha; NSAIDs, Non-Steroidal Anti-Inflammatory Drugs; csDMARDs, Conventional Synthetis Disease-Modifying Antirheumatic Drugs; Lp(a), Lipoprotein(a); ApoA1, Apolipoprotein A1; ApoB, Apolipoprotein B; IL, Interleukin; JAK, Janus Kinase; MACE, Major Adverse Cardiovascular Events; IMIDs, Immune-Mediated Inflammatory Diseases.
